# Antioxidant and food additive BHA prevents TNF cytotoxicity by acting as a direct RIPK1 inhibitor

**DOI:** 10.1038/s41419-021-03994-0

**Published:** 2021-07-14

**Authors:** Tom Delanghe, Jon Huyghe, Seungheon Lee, Dario Priem, Samya Van Coillie, Barbara Gilbert, Sze Men Choi, Peter Vandenabeele, Alexei Degterev, Gregory D. Cuny, Yves Dondelinger, Mathieu J. M. Bertrand

**Affiliations:** 1 VIB Center for Inflammation Research, 9052 Ghent, Belgium; 2 Department of Biomedical Molecular Biology, Ghent University, 9052 Ghent, Belgium; 3 Department of Pharmacological and Pharmaceutical Sciences, College of Pharmacy, University of Houston, Houston, TX 77204 USA; 4 Department of Developmental, Molecular & Chemical Biology, Tufts University School of Medicine, Boston, MA 02111 USA

**Keywords:** Kinases, Apoptosis, Necroptosis, Target identification, Cell death and immune response

## Abstract

Butylate hydroxyanisole (BHA) is a synthetic phenol that is widely utilized as a preservative by the food and cosmetic industries. The antioxidant properties of BHA are also frequently used by scientists to claim the implication of reactive oxygen species (ROS) in various cellular processes, including cell death. We report on the surprising finding that BHA functions as a direct inhibitor of RIPK1, a major signaling hub downstream of several immune receptors. Our in silico analysis predicts binding of 3-BHA, but not 2-BHA, to RIPK1 in an inactive DLG-out/Glu-out conformation, similar to the binding of the type III inhibitor Nec-1s to RIPK1. This predicted superior inhibitory capacity of 3-BHA over 2-BHA was confirmed in cells and using in vitro kinase assays. We demonstrate that the reported protective effect of BHA against tumor necrosis factor (TNF)-induced necroptotic death does not originate from ROS scavenging but instead from direct RIPK1 enzymatic inhibition, a finding that most probably extends to other reported effects of BHA. Accordingly, we show that BHA not only protects cells against RIPK1-mediated necroptosis but also against RIPK1 kinase-dependent apoptosis. We found that BHA treatment completely inhibits basal and induced RIPK1 enzymatic activity in cells, monitored at the level of TNFR1 complex I under apoptotic conditions or in the cytosol under necroptosis. Finally, we show that oral administration of BHA protects mice from RIPK1 kinase-dependent lethality caused by TNF injection, a model of systemic inflammatory response syndrome. In conclusion, our results demonstrate that BHA can no longer be used as a strict antioxidant and that new functions of RIPK1 may emerge from previously reported effects of BHA.

## Introduction

Butylated hydroxyanisole (BHA) and the structurally related lipophilic compounds butylated hydroxytoluene (BHT) and tert-butylhydroquinone (TBHQ) are commonly used food additives, with respective reference numbers E320, E321, and E319. These compounds are also frequently found in cosmetics, including skin lotions, shampoos, and lipsticks [1, 2]. The industrial interest for these synthetic phenols relies on their potent antioxidant properties, which prevent oxidation of fatty acids and thereby restrain rancidity, from which undesirable odors and flavors can result. The aromatic ring found in BHA, BHT, and TBHQ is able to stabilize free-radical reactive oxygen species (ROS) by sequestering them. Indeed, these compounds function as ROS scavengers by donating labile hydrogen to oxygen radicals derived from fatty acids, leaving an oxidized phenolic ion that is then stabilized by the resonance of the benzene ring [3]. Besides their wide use in industry, scientists have also shown interest in these aromatic compounds as easy tools to demonstrate the implication of cellular ROS in the regulation of specific signaling pathways. This is, for instance, the case in the TNF pathway, where several groups used BHA to claim the requirement of ROS for TNF-induced necroptosis [4–7], a regulated form of necrosis implicated in the host defense against viral infection, but also in the pathology of various diseases, including acute kidney injury and cardiac ischemia/reperfusion [8, 9].

TNF is well established as a major driver of inflammation and is consequently also a pharmacological target in several inflammatory disorders. TNF directly promotes inflammation by activating the MAPK and NF-κB signaling pathways, which collectively lead to the transcriptional upregulation of pro-inflammatory genes. But TNF can also indirectly promote inflammation by triggering cell death, in the form of extrinsic apoptosis or necroptosis. Death is, however, not the default response of TNF sensing. Engagement of TNFR1 by TNF induces the rapid recruitment of RIPK1 and TRADD to the cytosolic portion of the receptor, allowing the assembly of a primary membrane-bound multiprotein complex, known as TNFR1 complex I, which predominantly drives activation of the NF-κB and MAPK signaling pathways [10]. Cell death instead requires the inactivation of specific cell death checkpoints in the pathway [11–13], and the subsequent formation of a receptor-dissociated FADD-containing secondary cytosolic complex termed complex IIa, IIb, or necrosome, depending on its composition. While complex IIa/b triggers caspase-8-mediated apoptosis, the necrosome assembles in caspase-8 inhibited conditions and promotes RIPK3/MLKL-dependent necroptosis [5, 14–20]. Execution of necroptosis relies on the activation of RIPK3 and subsequent phosphorylation of MLKL by RIPK3, which induces a conformational change in MLKL. Ultimately, phosphorylated MLKL translocates to the plasma membrane where it either directly or indirectly causes plasma membrane rupture [17, 18, 20, 21]. The enzymatic activity of RIPK1 is dispensable for complex I and complex IIa assembly but is required for the formation of complex IIb and of the necrosome [22–24]. Depending on the cellular context, the catalytic activity of RIPK1 can therefore promote apoptosis or necroptosis downstream of TNFR1.

Several groups showed that BHA protects cells from TNF-induced RIPK1 kinase-dependent necroptosis, implying that ROS would be required for the execution of this cell death modality [4–7]. An assumption that was further motivated by the fact that ROS is readily detected in cells undergoing TNF-induced necroptosis, through a process reported to rely on TRAF2, FADD, RIPK1, and RIPK3 [4, 6, 7, 17, 19, 25]. The cellular source of the pro-necroptotic ROS and the exact mechanism by which these radicals would promote necroptosis have, however, remained unclear. Indeed, although most studies refer to a role for mitochondrial ROS [4, 5, 17, 19, 25, 26], a few others proposed that the killing radicals would instead be generated by the NADPH oxidase complexes 1 and 2, which are recruited to TNFR1 upon TNF sensing [27, 28]. This last notion may be supported by the fact that cells depleted of mitochondria are still able to undergo necroptosis [29], questioning the importance of the mitochondria [30], but also of ROS generation, for necroptosis induction. Although it is unclear how ROS would promote necroptosis, their inhibition by BHA was shown to prevent necrosome assembly, thereby positioning their potential mode of action upstream of the cytosolic death complex [22, 31]. One study further proposed a direct link between ROS generation and RIPK1 activation. In that study, the authors report oxidation of RIPK1 on specific cysteine residues as a prerequisite for its enzymatic activation, and for the subsequent necrosome assembly [26]. However, the requirement of ROS for RIPK1 kinase-dependent necroptosis has also been contested, in part by showing that pretreatment of cells with antioxidants other than BHA does not protect cells from TNF-induced necroptosis [32, 33]. In line with this, the anti-necroptotic potential of BHA was even proposed to surpass its ROS-scavenging properties by affecting mitochondrial complex I and lipoxygenases activities [34]. To date, it, therefore, remains unclear whether ROS is a side product or a necessary means for TNF-induced necroptosis.

In this study, we made the surprising finding that BHA acts as a direct RIPK1 inhibitor, and we demonstrate that the anti-necroptotic effect of BHA is a result of direct RIPK1 inhibition and not ROS depletion. Accordingly, we show that BHA not only protects cells against RIPK1-mediated necroptosis but also against RIPK1 kinase-dependent apoptosis. In line with this, RIPK1-independent cell death by ZBP1-mediated necroptosis or chemotherapeutic apoptosis is not affected by BHA, demonstrating that targeting of RIPK1 by BHA is its most prominent effect in cell death. Finally, we show that oral administration of BHA prevents in vivo inflammatory conditions driven by TNF-mediated RIPK1 kinase-dependent cell death, which may have beneficial implications for its use as a food additive.

## Material and methods

### Antibodies and reagents

The following antibodies were used throughout this manuscript: anti-RIPK1 (BD Biosciences #610459, 1:2000; Cell Signaling #3493, 1:2000); anti-IκBα (Santa Cruz sc-371, 1:2000); anti-actin (MP Biomedicals #69100, 1:20000); anti-β-tubulin-HRP (Abcam ab21058, 1:10000); anti-pSer166 human RIPK1 (Cell Signaling #65746, 1:1000), anti-TRADD (Bio-Rad AHP2533, 1:1000), anti-Caspase-8 (Abnova MAB3429 clone 1G12, 1:1000), anti-FADD (Enzo ADI-AAM-212-E, 1:1000). The anti-pSer166/T169 mouse RIPK1 is a custom-made rabbit polyclonal antibody produced by ThermoFisher Scientific following the 2-rabbit 90-day protocol, as previously described [35]. The following commercially available recombinant proteins were used: mouse and human TNF (used concentrations indicated in the figure legends) and FLAG-tagged human TNF (1 µg/ml) were purchased from the VIB Protein Service Facility (Ghent, Belgium); murine IFNβ (Enzo Life Sciences, 100 U/ml); lambda protein phosphatase (New England Biolabs P0753s). The following compounds were used: TPCA-1 (IKKi) (Tocris Bioscience, 5 µM); GSK8612 (TBK1i) (MedChemExpress, 10 µM); GSK’872 (RIPK3i) (Selleckchem, 3.3 µM); zVAD-fmk (Bachem, 50 µM); Cycloheximide (CHX) (Sigma-Aldrich, 0.5 µg/ml); Nec-1s (RIPK1i, UAMC-02197)(Laboratory of Medicinal Chemistry, University of Antwerp, Belgium, 10 µM); Etoposide (SelleckChem, 10 µM); Staurosporine (Sigma-Aldrich, 2 µM); ML162 (Aobious Inc., 5 µM); Erastin (SelleckChem, 10 µM); BHA and BHT (Sigma-Aldrich, 100 µM unless otherwise stated); 2-BHA (BLD Pharmatech Co., 100 µM unless otherwise stated); 3-BHA (Combi-Blocks, 100 µM unless otherwise stated); TBHQ (Sigma-Aldrich, 100 µM); *N*-Acetyl-l-cysteine (NAC) (Sigma-Aldrich, 5 mM); α-tocopherol and its water soluble counterpart Trolox (Sigma-Aldrich, 100 µM); DecylQ (Sigma-Aldrich, 10 µM); Ferrostatin-1 (Fer-1) (Matrix Scientific, 500 nM); Dabrafenib (SelleckChem, 10 µM); Rotenone (Sigma-Aldrich, 25 µM), NDGA (Sigma-Aldrich, 100 µM).

### Cell lines

Mouse embryonic fibroblasts (MEFs) were isolated from C57Bl6/J E12.5 embryos according to standard protocol and immortalized by transfection of an SV40 large T-expressing construct. *Ripk3*^*+/+*^ and *Ripk3*^*−/−*^ MEFs have been isolated from littermate embryos of *Ripk3*^*+/−*^ pregnant females [36] and have been described previously [37]. MEFs, mouse dermal fibroblasts (MDFs), L929 cells, and human breast cancer cell line BT549 were cultured in Dulbecco’s modified Eagle’s medium supplemented with 10% fetal calf serum, l-glutamine (200 mM), sodium pyruvate (400 mM), penicillin (100 IU/ml), and streptomycin (0.1 mg/ml) in normoxic conditions (5% CO_2_). HT-29 cells were cultured in McCoys modified medium supplemented with 10% fetal calf serum, l-glutamine (200 mM), sodium pyruvate (400 mM), penicillin (100 IU/ml), and streptomycin (0.1 mg/ml) in normoxic conditions (5% CO_2_). All cell lines were routinely tested for mycoplasma. Only the HT-29 cells were authenticated.

### Cell death assays

The cells were seeded in duplicates or triplicates in a 96-well plate (MEFs: 15,000/well). The next day, the cells were pretreated with the indicated compounds for 30 min and then stimulated with the indicated concentration of hTNF in the presence of 5 μM SytoxGreen (Invitrogen). SytoxGreen intensity was measured at intervals of 1 h using a Fluostar Omega fluorescence plate reader, with an excitation filter of 485 nm, an emission filter of 520 nm, gains set at 1100, 20 flashes per well, and orbital averaging with a diameter of 3 mm. The percentage of cell death was calculated as follows: (induced fluorescence-background fluorescence)/(max fluorescence-background fluorescence)×100. The maximal fluorescence is obtained by full permeabilization of the cells by using Triton X-100 at a final concentration of 0.1%. All cell death data are presented as mean ± SEM of n (indicated in the Figure) independent experiments unless stated otherwise.

### HSV infection assay

MEFs were seeded in triplicates in a 96-well plate (10,000/well) in the morning, and pretreated in the afternoon with 100 U/ml IFNβ for 24 h. The cells were subsequently infected with ICP6 RHIM mutant Herpes Simplex Virus 1 F*mut*strain [38] with a multiplicity of infection of 3 in the presence of 2.5 µM SytoxGreen (Invitrogen). SytoxGreen intensity was measured at intervals of 1 h using an in‐incubator imaging platform (IncuCyte ZOOM, Essen Bioscience; ×10 objective, one scan per well in 96‐well plates per hour; samples were imaged in triplicate wells).

### Immunoprecipitations

For the TNFR1 complex I (CI) IPs, Complex IIb/necrosome IPs (CII IPs), and pS166/T169 IPs, 7.5 × 10^6^ cells were seeded per condition in a 140 cm² petri dish. The next day, the cells were pretreated as indicated in the figure legends and subsequently stimulated (or not) with 1 µg/ml FLAG-hTNF (CI IPs), 20 ng/ml hTNF (CII IPs), or 1 µg/ml hTNF (pS166/T169 IPs) for the indicated period of time. The cells were then washed twice in ice-cold PBS and lysed in 1 ml NP-40 lysis buffer (10% glycerol, 1% NP-40, 150 mM NaCl and 10 mM Tris-HCl pH 8 supplemented with phosphatase and protease inhibitor cocktail tablets (Roche Diagnostics)). The cell lysates were cleared by centrifugation at 21,000 × *g* for 10 min at 4°C and the supernatants were then incubated overnight with FLAG M2 affinity gel (Sigma-Aldrich)(CI IPs), Caspase-8 antibody coupled to protein G beads (CII IPs) or pS166/T169 RIPK1 antibody coupled to protein G beads (pS166/T169 IPs). The next day, the beads were washed three times in NP-40 lysis buffer at 4°C. When indicated, the immunoprecipitated protein complexes were additionally deubiquitylated by USP21 and dephosphorylated by λ protein phosphatase. To do so, the beads were resuspended after the final wash in 50 µL 1× DUB/λPP buffer (50 mM Tris-HCl pH 8, 50 mM NaCl, 5 mM DTT and 1 mM MnCl_2_) together with 1.2 µg USP21 (homemade) and 400 U λ PPase. Enzymatic reactions were allowed to proceed for 30 min at 37°C. During USP21 or λ PPase treatment, 150 ng/ml 3× FLAG-peptide was added to the reaction mixture to release complex I from the FLAG M2 affinity gel (CI IPs). The eluted complex was subsequently collected and diluted to 1× Laemmle buffer for direct immunoblot analysis.

### Production of recombinant proteins

Recombinant human RIPK1 (AA 1–479) and mouse RIPK3 (AA 1–439) were produced in Sf9 insect cells as GST-fusion protein, as previously described [35]. In briefly, protein-GST-fusion constructs were obtained by cloning the respective cDNA for RIPK1 and RIPK3 into the pAcGHLT vector (BD Biosciences). Baculovirus was obtained after co-transfection of these constructs with ProEasy linearized baculovirus (AB Vector) into Sf9 cells according to the manufacturer’s instructions. Sf9-cell pellets were resuspended in 20 mM Tris-HCl pH 8.0, 200 mM NaCl, 1 mM EDTA, 0.5% (v/v) Igepal CA-630, EDTA-free Protease Inhibitor Cocktail Tablets (Roche Diagnostics). Lysates were incubated on ice for 30 min. Insoluble proteins were removed by centrifugation at 10,000 × *g* for 15 min. The supernatant was applied to a Glutathione Sepharose 4FFcolumn (GE Healthcare) pre-equilibrated with PBS pH 7.4. The GST-tagged RIP kinases were eluted from the column with 50 mM Tris-HCl pH 8.0, 100 mM NaCl, 15 mM reduced glutathione. Fractions containing the RIP kinases were pooled and further purified using a Superdex 75 pg column (GE Healthcare, running buffer: 20 mM Tris-HCl pH 8.0, 100 mM NaCl). The purity of the fractions was checked by means of sodium dodecyl sulfate–polyacrylamide gel electrophoresis (SDS-PAGE), the RIP kinase fractions were pooled. In all, 10% glycerol and 5 mM DTT were added to the protein fraction, followed by storage at −70°C.

pLH36-hUSP21 (AA 195-565) plasmid was transformed in *Escherichia coli* strain MC1061 containing the pICA2 plasmid, which allows IPTG-inducible expression. Exponentially growing cultures (28°C) were induced with 0.5 mM IPTG and incubated overnight at 20 °C. Cell pellets were resuspended in buffer A (50 mM Tris-HCl pH 7.4; 300 mM NaCl, DNase I (1 mg/100 mL) (Roche Diagnostics) and complete, EDTA-free Protease Inhibitor Cocktail Tablets (Roche Diagnostics)), and lysed by sonication. Insoluble proteins were removed by centrifugation (38,000 × *g* for 1 h). The supernatant in presence of 10 mM imidazole was applied to a nickel HisTrap HP columns (GE Healthcare) pre-equilibrated with buffer B (50 mM Tris-HCl pH 7.4; 300 mM NaCl; 10 mM imidazole). His_6_-hUSP21 was eluted from the column with buffer C (50 mM Tris-HCl pH 7.4; 50 mM NaCl; 200 mM imidazole). Elution fractions containing His_6_-hUSP21 were pooled and desalted on a Sephadex G-25 column (GE Healthcare) against buffer D (25 mM Tris-HCl pH 7.4; 100 mM NaCl). The His_6_-hUSP21 fusion protein was digested with His_6_-caspase-3 to clip off His_6_-tag. The digested sample was run on a nickel HisTrap HP columns (GE Healthcare) pre-equilibrated with buffer E (50 mM Tris-HCl pH 7.4; 300 mM NaCl; 10% glycerol) for removal of the His_6_-tag and His_6_-caspase-3. hUSP21 was eluted from the column with buffer F (50 mM Tris-HCl pH 7.4; 300 mM NaCl; 10% glycerol; 75 mM imidazole). Elution fractions containing hUSP21 were pooled. The purified recombinant hUSP21 protein was dialyzed to buffer G (25 mM Tris-HCl pH 7.4; 100 mM NaCl; 10% glycerol; 5 mM DTT). The purity of the fractions was checked by means of SDS-PAGE.

### Kinase assays

Quantitative in vitro kinase assays were performed by using the ADP-Glo kinase assay kit (Promega). In brief, recombinant human RIPK1 (300 ng/reaction) or mouse Ripk3 (30 ng/reaction) was incubated for 4 h at room temperature in kinase assay buffer (50 µm ATP, 25 mM HEPES pH 7.5, 25 mM NaCl, 15 mM MgCl_2_, 0.25 mg/ml BSA, 0.01% CHAPS and 2 mM DTT) in the presence or absence of the compounds. To convert ATP consumption into light production, a 2:2:1 (kinase assay reaction:ADP-Glo reagent:kinase detection reagent) ratio of the kit’s components was used. Luminescence was measured during 1 s reads with the GloMax 96 microplate luminometer (Promega).

### Molecular docking studies

Molecular docking studies were performed using molecular operating environment (MOE). Ligands were created using ChemDraw 3D Bio and subjected to energy minimization using MM2 force field. RIPK1 protein was extracted from an available co-crystal structure of RIPK1 (PDB: 4ITH) using BIOVIA Discovery Studio Visualizer 2016 software (http://www.3dsbiovia.com). Monomer subunit B of RIPK1 was eliminated from the dimer as well as the iodide ion, sodium ion, water molecules, and Nec-1s. The standard protocol of MOE was used and binding pockets by site finder were selected to include potential interactions that were observed in the DLG-out/Glu-out Nec-1s•RIPK1 complex. The docking results were analyzed using Pymol visualization software (http://pymol.org). Binding poses were selected that demonstrated similar interactions compared with Nec-1s and consistency of poses with low binding scores. Similarly, RIPK1 protein was extracted from an available DLG-out/Glu-in co-crystal structure of RIPK1 (PDB: 4NEU). The monomer subunit A of RIPK1 was eliminated from the dimer as well as the water molecules and the 1-aminoisoquinoline inhibitor. Again, the standard protocol of MOE was used and binding pockets by site finder were selected to include potential interactions that were observed in the 1-aminoisoquinoline derivative RIPK1 complex. The docking results were analyzed using Pymol.

### TNF-induced shock model

In all, 10-week old C57Bl6/J female mice (Janvier-Labs) were intravenously (i.v.) injected with 15 µg mTNF (VIB Protein Service Facility, Ghent, Belgium) diluted in endotoxin-free PBS pH6.8 per 20 g of body weight. Mice were starved for 16 h before the mTNF injection to minimize variation on the uptake of the compounds via oral gavage. Oral gavage was performed 1 h before mTNF injection with either corn oil or 625 mg/kg BHA/BHT in corn oil. Mortality and body temperature were monitored until 3 days after mTNF injection. Rectal body temperature was recorded with an industrial electric thermometer (Comark Electronics, Norwich, UK; model 2001). The humane endpoints were described in the EC (EC2017–079, Ghent University). Mice were killed as soon as their body temperature dropped below 25°C or when they showed visible convulsions.

### *Sharpin*^*cpdm/cpdm*^ mice fed on BHA/BHT-containing diets

The *Sharpin*^*cpdm/cpdm*^ mice were described earlier [38]. At 4-week of age, *Sharpin*^*cpdm/cpdm*^ and *Sharpin*^*+/+*^ littermates were fed a standard diet enriched, or not, with 0.7% w/w BHA or BHT (Ssniff, Soest, Germany). After 5 weeks on this diet, the mice were killed and the degree of inflammatory symptoms was assessed. The organs were fixed in 4 % paraformaldehyde, embedded in paraffin, and cut at 3 or 5 µm thickness. Subsequently, sections were stained with hematoxylin and eosin. The terminal deoxynucleotidyl transferase dUTP nick end labeling assay was performed according to the manufacturer’s instructions (In situ cell death detection kit, TMR red—Roche). Micrographs were acquired using a Zeiss Axioscan Z.1 slide scanner (Carl Zeiss, Jenna, Germany) at ×20, ×100, ×200, and ×400 magnification, with a Hamamatsu ORCA Flash4 camera (Hamamatsu Photonics) or AxioCam MRm Rev. 3 FireWire camera, via either Zen 3.1 software or AxioVision 4.5 software from Zeiss. Quantification analysis was performed using a script provided by the VIB Bioimaging Core (Ghent, Belgium) ran on QuPath-0.2.3 software. Serum LDH levels were obtained at UZ-Gent (Belgium) using Cobas 8000 modular analyzer series (Roche Diagnostics, Basel, Switzerland). Interleukin (IL)-6 levels were measured using a Bio-Plex Multiplex immunoassay (Bio-Rad #171304070) according to the manufacturer’s instructions.

### Statistical analysis

Statistical analyses were performed with GraphPad Prism V9 (https://www.graphpad.com/scientific-software/prism) and the type of analysis is specified in the figure legends with the exception of kinetic cell death experiments with more than one timepoint and more than two conditions that do not have a routine analysis available in Graphpad Prism V9. These experiments were analyzed as follows: Cell death values by Sytox Green positivity (SG^+^) were analyzed as repeated measurements using the method of residual maximum likelihood, as implemented in Genstat for Windows 21^st^ edition. In brief, a linear mixed model (random terms underlined) of the form *y* = experiment + treatment + time + treatment × time + subject × time was fitted to the longitudinal data. The term subject × time represents the residual error term with dependent errors because the repeated measurements are taken in the same subject, causing correlations among observations. Several covariance models were fitted to the data to account for the correlation present in the data. The autoregressive correlation model of order 1 (AR1) was finally selected as the best-fitted model based on Akaike’s information criterion coefficient. The AR covariance model assumes that correlation between observations decays as the measurements are collected further apart in time. Additional options selected to get a best-fitting model included (1) times of measurement were set as equally spaced, and (2) allowance of unequal variances across time. The significance of the fixed terms in the model and significance of changes in the difference between treatment effects over time was assessed using an approximate *F* test as implemented in Genstat for Windows 21st edition.

For the in vivo experiments, the sample size was predetermined using the Gpower 3.1 software to detect a pre-specified effect size. Animals were randomly assigned to a treatment group and no blinding was performed for the assignment. Researchers were blinded for the analysis of the data (cytokines, microscopy, lethality monitoring).

## Results

### BHA specifically protects cells from RIPK1 kinase-dependent cell death

**Fig. 1:**
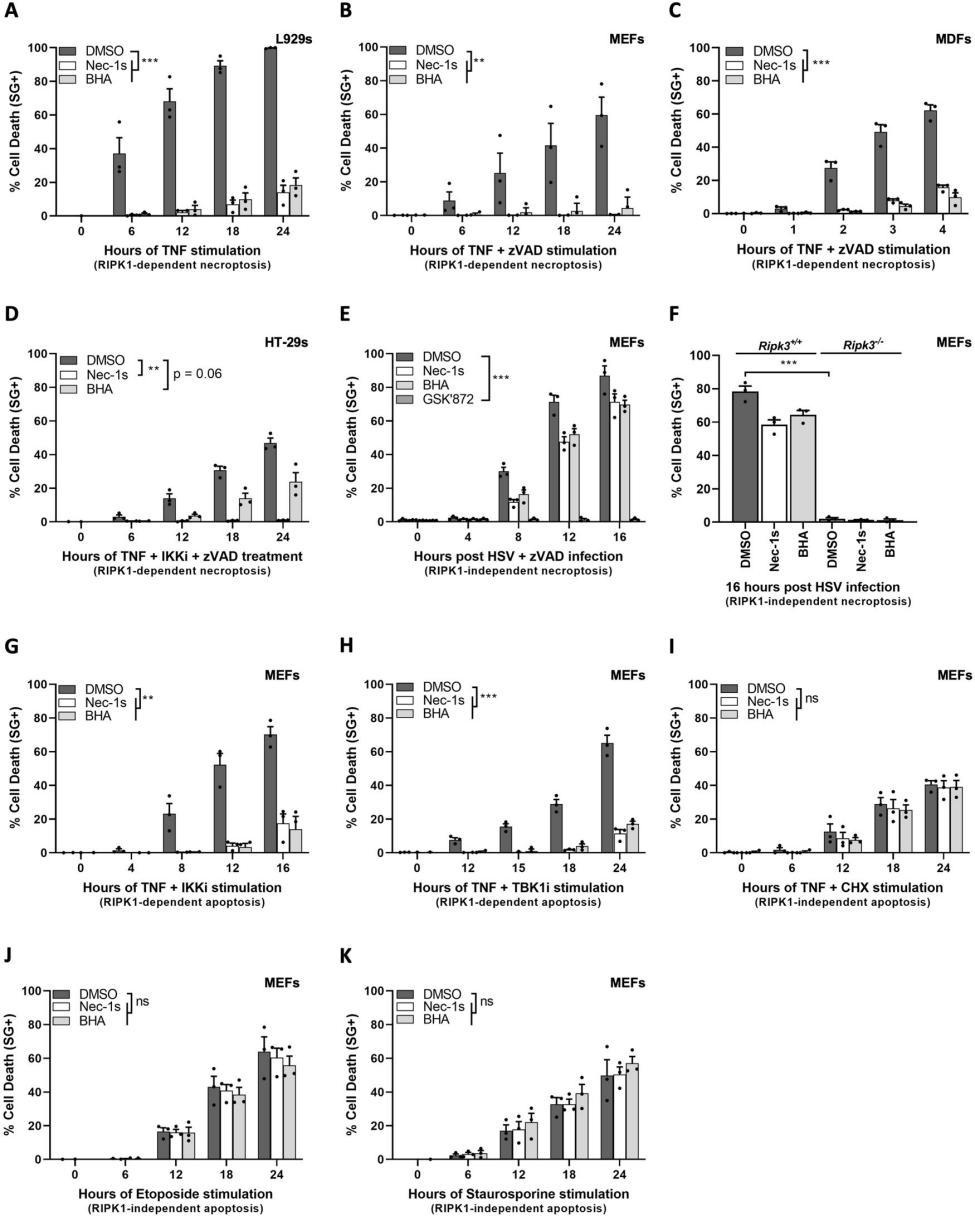
BHA specifically protects cells from RIPK1 kinase-dependent cell death. **A**–**D**, **G**–**K** L929 cells (**A**), MEFs (**B**, **G**–**K**), MDFs (**C**), and HT-29 cells (**D**) were pretreated for 30 min with indicated compounds (100 µM BHA, 10 µM Nec-1s, 500 ng/ml CHX) before stimulation with 20 ng/ml hTNF (**A**–**D**, **G**–**I**), 10 µM etoposide (**J**) or 2 µM staurosporine (**K**) for the indicated duration. **E**–**F** MEFs of indicated genotypes were pretreated for 24 h with 100 U/ml IFNβ followed by pretreatment for 30 min with indicated compounds and infected with ICP6 RHIM mutant HSV1 fmutRHIM with an MOI of 3. Cell death was measured over time by Sytox Green (SG+) positivity. Cell death assays are presented as mean ± SEM of three independent experiments (*n* = 3). Statistical analysis on kinetic cell death assays with more than one timepoint is detailed in the Methods section. Significance between samples is indicated in the figures as follows: *P < 0.05; **P < 0.01; ***P < 0.001; NS, not significant.

TNF triggers necroptosis in mouse L929 cells upon single exposure (Fig. 1A), in MEFs and dermal fibroblasts (MDFs) by the additional presence of the pan-caspase inhibitor zVAD-fmk (Fig. 1B, C), and in human HT-29 cells by the further inhibition of IKKα/β (IKKi) (Fig. 1D). In all four cellular systems, the requirement of RIPK1 kinase activity for necroptosis induction can be demonstrated by the use of the RIPK1 inhibitor Nec-1s, which completely protects the cells from TNF cytotoxicity. In line with previous studies, we found that BHA pretreatment also greatly protected these cells from TNF-induced necroptosis (Fig. 1A–D). However, we found that BHA did not provide protection against necroptosis that does not rely on the kinase activity of RIPK1, as observed following infection of MEFs by Herpes simplex virus 1 (HSV1) [38] (Fig. 1E, F). In these experiments, the cells are pretreated for 24 h with IFNβ to induce expression of ZBP1, and are subsequently infected with a mutated strain of HSV1 (ICP6 RHIM mutant) to specifically induce ZBP1/RIPK3-dependent necroptosis [39]. As shown in Fig. 1E, F, Nec-1s and BHA similarly and marginally protected these cells from death, whereas pharmacological (Fig. 1E) and genetic (Fig. 1F) inhibition of RIPK3 completely prevented necroptosis induction. Of note, zVAD-fmk was added to the experiment making use of the RIPK3 inhibitor GSK’872 to prevent spontaneous induction of apoptosis [40].

Interestingly, we found that the correlation between the anti-death potential of BHA and the dependency on RIPK1 kinase activity was also true in the context of apoptosis. TNF triggers RIPK1 kinase-dependent apoptosis when combined with IKKα/β [35, 41] (IKKi) (Fig. 1G) or IKKε/TBK1 [42, 43] (TBK1i) inhibition (Fig. 1H), and RIPK1-independent apoptosis in the presence of the translational inhibitor CHX [44] (Fig. 1I). Remarkably, we observed perfect overlap in the protection provided by Nec-1s and BHA in cells undergoing TNF-induced RIPK1 kinase-dependent apoptosis (Fig. 1G, H). In addition, BHA had no effect against RIPK1-independent apoptosis induced by TNF in a combination of CHX (Fig. 1I), and by etoposide (Fig. 1J) or staurosporine (Fig. 1K) treatment.

Together, these results demonstrated that the protective effect of BHA is not specific to necroptosis but rather to RIPK1 cytotoxicity, which suggested a role for ROS in RIPK1 activation, as previously reported [26].

### The protective role of BHA does not originate from ROS scavenging

**Fig. 2:**
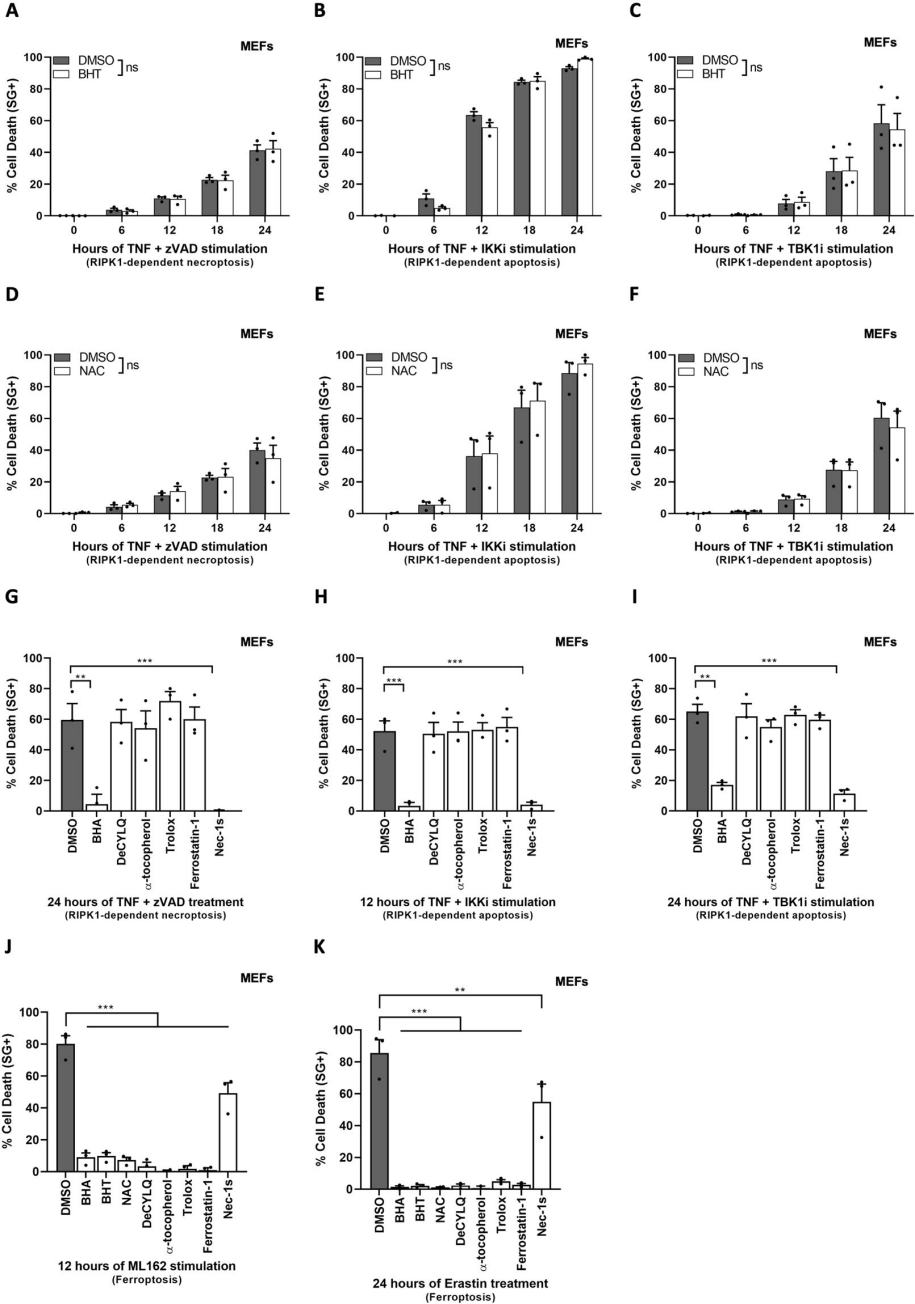
The protective role of BHA is independent of its ROS-scavenging properties. **A**–**K** MEFs were pretreated for 30 min with indicated compounds (100 µM BHT, 5 mM NAC, 100 µM BHA, 10 µM DecylQ, 100 µM α-tocopherol, 100 µM Trolox, 500 nM Ferrostatin-1, 10 µM Nec-1s) before stimulation with 20 ng/ml hTNF (**A**–**I**), 5 µM ML162 (**J**), or 10 µM Erastin (**K**) for the indicated duration. Cell death was measured over time by Sytox Green (SG+) positivity. Cell death assays are presented as mean ± SEM of three independent experiments (*n* = 3). **A**–**F** Statistical analysis on kinetic cell death assays with more than one timepoint is detailed in the Methods section. **G**–**K** Statistical significance was determined via ordinary one-way ANOVA followed by a Tukey post hoc test. Significance between samples is indicated in the figures as follows: *P < 0.05; **P < 0.01; ***P < 0.001; NS, not significant.

In order to evaluate whether ROS has an essential role during RIPK1 kinase-dependent death, we tested the effect of other ROS scavengers on TNF-induced RIPK1 cytotoxicity, starting with the structurally related synthetic compound BHT. Interestingly, and in contrast to BHA, pretreatment with BHT did not protect MEFs, MDFs, or L929 cells from TNF-induced necroptosis (Fig. 2A, SFig. 1A–B), nor from TNF-induced RIPK1 kinase-dependent apoptosis (Fig. 2B, C). The absence of protection was also noticed in the presence of five additional antioxidants, including the hydrophilic ROS scavenger NAC (Fig. 2D–F), the ubiquinone analog and membrane-targeted ROS scavenger DeCylQ, the vitamin E isomer, and natural antioxidant α-tocopherol, its water-soluble counterpart Trolox and finally the lipid ROS scavenger Ferrostatin-1 (Fig. 2G–I). Importantly, all these antioxidants were in contrast equally efficient at preventing ferroptosis, a ROS-dependent cell death modality, induced by GPX4 inhibition via ML162 treatment or by system xc-inhibition following erastin exposure [45, 46] (Fig. 2J, K). These results demonstrated that ROS is dispensable for TNF-induced RIPK1 kinase-dependent apoptosis and necroptosis, and thereby questioned the origin of the protective effect of BHA against RIPK1 cytotoxicity.

### BHA prevents cellular activation of RIPK1

A previous study reported that the anti-necroptotic potential of BHA was partially due to its ability to inhibit the activity of mitochondrial complex I and of lipoxygenases, which was supported by the protection against TNF-induced necroptosis obtained by their respective inhibition with rotenone and NDGA [34]. We found that the protection conferred by rotenone and NDGA likely originates from defective TNFR1 complex I assembly, as monitored by defective TRADD and RIPK1 recruitment to TNFR1 (SFig. 2A), which consequently affects global signaling from the receptor, as exemplified by defective IκBα degradation (SFig. 2A). The fact that BHA does not affect complex I assembly, therefore, indicates that BHA protects cells from TNF-mediated RIPK1 cytotoxicity through a distinct mechanism (SFig. 2B).

**Fig. 3:**
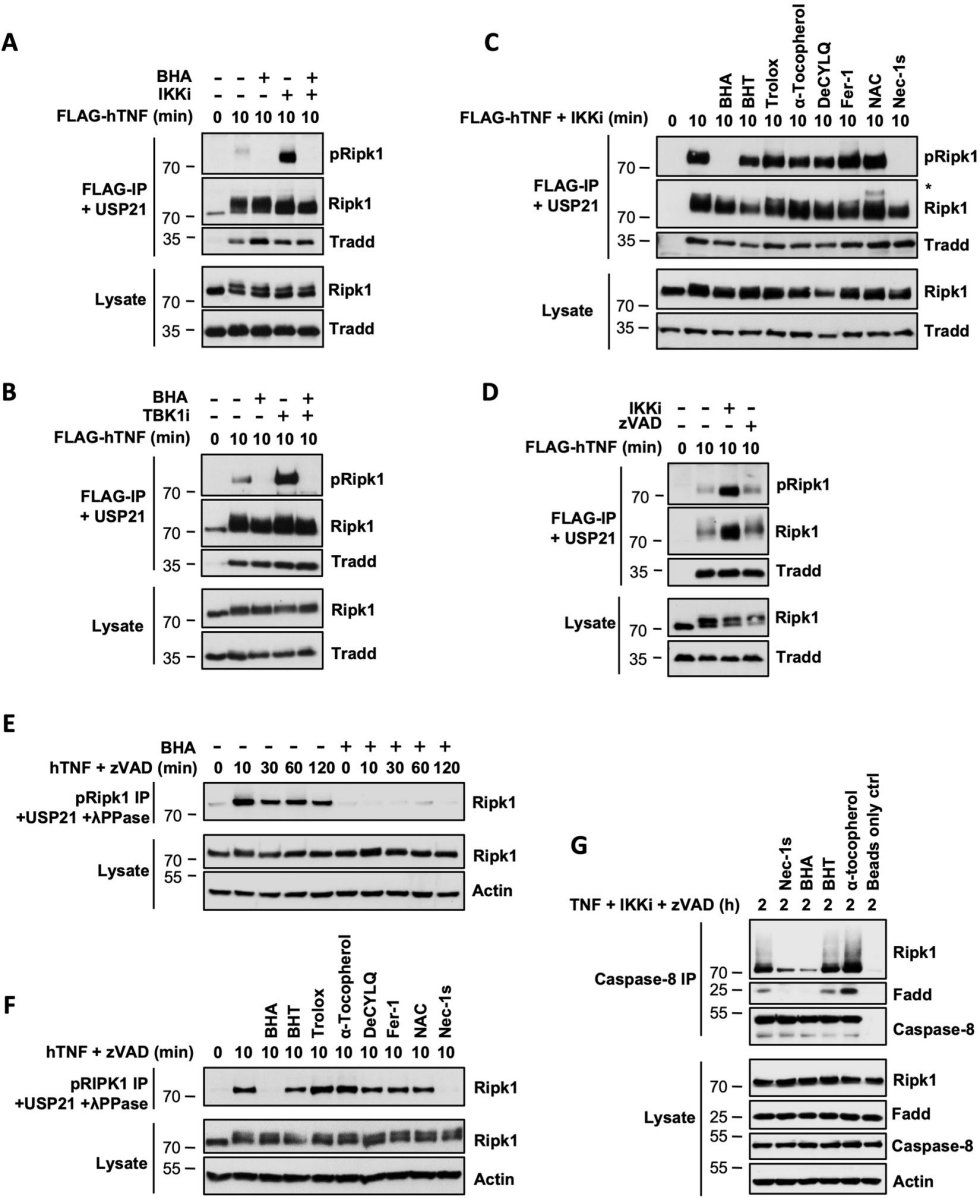
BHA prevents cellular activation of RIPK1. **A**–**G** MEFs were pretreated for 30 min with the indicated compounds (100 µM BHA, 100 µM BHT, 5 mM NAC, 10 µM DecylQ, 100 µM α-tocopherol, 100 µM Trolox, 500 nM Ferrostatin-1, 10 µM Nec-1s, 5 µM TPCA-1 (IKKi), 10 µM GSK8612 (TBK1i), 50 µM zVAD-fmk) before stimulation with 1 µg/ml FLAG-hTNF (**A**–**D**), 1 µg/ml hTNF (**E**–**F**) or 20 ng/ml hTNF (**G**) for the indicated duration. **A**–**D** TNFR1 complex I was FLAG-immunoprecipitated and the IPs were treated with USP21 before analysis by immunoblot. The signal for pRIPK1 refers to active RIPK1 autophosphorylated on residue S166/T169. **E**–**F** Autophosphorylated active RIPK1 (pRIPK1) was immunoprecipitated using the specific anti-pS166/T169 RIPK1 antibody and the IPs were treated with USP21 and λ phosphatase before analysis by immunoblot. **G** Complex IIb/necrosome was pulled down by immunoprecipitation of caspase-8 and analyzed by immunoblot. Immunoblots are representative of at least two independent experiments.

We, and others, previously reported an early boost in RIPK1 activity, monitored by autophosphorylation on S166/T169 [35, 42, 47, 48], at the level of TNFR1 complex I under conditions of RIPK1 kinase-dependent apoptosis. In these experiments, TNFR1 complex I is incubated with USP21 to remove the ubiquitin chains attached to RIPK1, allowing proper visualization of RIPK1 phosphorylation by immunoblot. Interestingly, we found that BHA pretreatment of MEFs and human HT-29 and BT549 cells not only prevented the massive activation of RIPK1 resulting from IKKα/β or IKKε/TBK1 inhibition, but also the basal activity detected at the receptor complex upon single TNF stimulation (Fig. 3A, B, SFig. 2C, D). We confirmed that this effect was specific to BHA as pretreatment with the panel of antioxidants did not alter RIPK1 activation in TNFR1 Complex I (Fig. 3C). Such an early boost in RIPK1 activity was not observed at the receptor complex upon stimulation of cells with the necroptotic trigger TNF + zVAD-fmk (Fig. 3D), but was instead detected, at around the same time, in a pool of cytosolic RIPK1 (Fig. 3E). In these experiments, immunoprecipitated pS166/pT169 RIPK1 was preventively incubated with USP21 and λ-phosphatase to limit the potential complexity of its migration profile by immunoblot. Remarkably, both BHA and Nec-1s again prevented RIPK1 activation under these necroptotic conditions (Fig. 3E, F), which was not observed with any of the other antioxidants (Fig. 3F). As the catalytic activity of RIPK1 is required for the formation of the secondary cytosolic complex IIb/necrosome that triggers RIPK1 kinase-dependent apoptosis and necroptosis, we finally confirmed that the early inhibition of RIPK1 by BHA translated into defective complex IIb/necrosome assembly. As shown in Fig. 3G, BHA and Nec-1s similarly prevented the RIPK1 kinase-dependent association between RIPK1, FADD, and Caspase-8 in response to TNF + IKKi+zVAD-fmk, an inhibition that was not observed by BHT or α-tocopherol pretreatment (Fig. 3G). Together, these results demonstrated that BHA protects cells from TNF-induced RIPK1 kinase-dependent cell death by inhibiting cellular RIPK1 enzymatic activity.

### BHA acts as a direct RIPK1 inhibitor

**Fig. 4:**
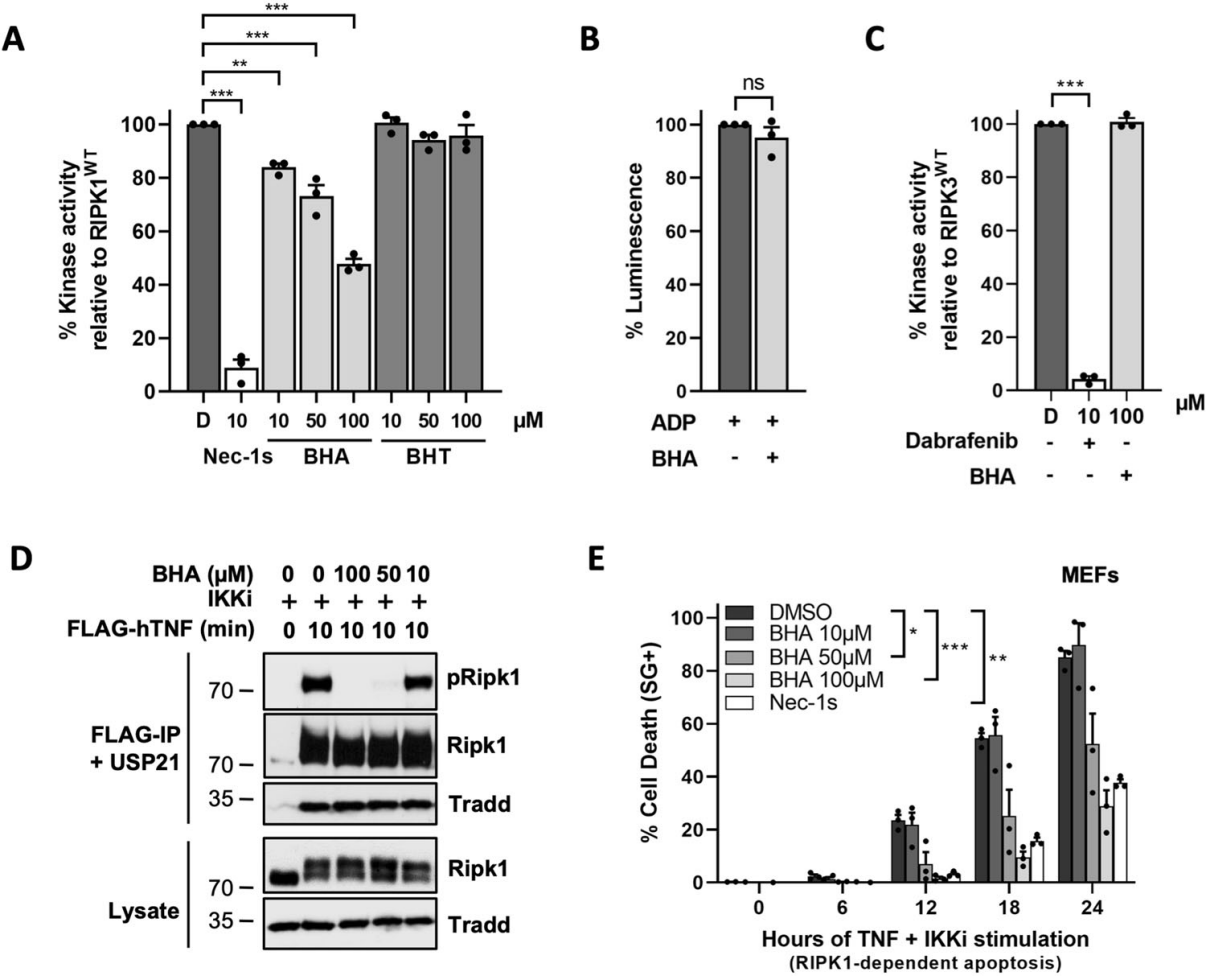
BHA acts as a direct RIPK1 kinase inhibitor. **A**, **C** Quantitative RIPK1 (AA 1–479) (**A**) or Ripk3 (AA 1–439) (**C**) enzymatic activities were measured by ATP consumption using ADP-Glo kinase assays. The compounds were used at indicated concentrations and ‘D’ is short for DMSO. **B** The ADP-Glo reaction was performed in the absence of active kinase to assess the possible interference of BHA (100 µM) with the luminescence reaction. Results are presented as a percentage relative to the activity of the RIPK1/3 in absence of inhibitors (**A**, **C**), or as a percentage relative to the ADP-Glo luminescence reaction (**B**) and are the mean ± SEM of three independent kinase assays (*n* = 3). Statistical significance was determined by one-way ANOVA followed by a Tukey post hoc test (**A**–**B**) or by a two-tailed paired *t* test (**C**). **D** MEFs were pretreated for 30 min with the indicated compounds (5 µM TPCA-1) before stimulation with 1 µg/ml FLAG-hTNF for the indicated duration. TNFR1 complex I was then FLAG-immunoprecipitated and the IPs were treated with USP21 before analysis by immunoblot, where pRIPK1 refers to autophosphorylation of RIPK1 on S166/T169. The results are representative of at least two independent experiments. **E** MEFs were pretreated for 30 min with the indicated compounds before stimulation with 20 ng/ml hTNF for the indicated duration. Cell death was measured over time by Sytox Green (SG+) positivity, and the results are presented as mean ± SEM of three independent experiments (*n* = 3). Statistical analysis on kinetic cell death assays with more than one timepoint is detailed in the Methods section (**E**). Significance between samples is indicated in the figures as follows: *P < 0.05; **P < 0.01; ***P < 0.001; NS, not significant.

We next tested the possibility that BHA would directly inhibit RIPK1 by performing in vitro kinase assays using recombinant RIPK1. Remarkably, we observed a dose-dependent inhibitory effect of BHA on RIPK1 (Fig. 4A), reaching a 50% reduction in RIPK1 enzymatic activity at 100 µM of BHA. Importantly, BHT had no effect on RIPK1 activity (Fig. 4A), and the inhibition by BHA was not originating from interference with the biochemical principle of the ADP-GLO^TM^ kinase assay, the conversion of ADP to light (Fig. 4B). In addition, the inhibitory effect of BHA showed specificity to RIPK1, as the enzymatic activity of the close family member RIPK3 was not affected by BHA, while completely repressed by the RIPK3 inhibitor Dabrafenib [49] (Fig. 4C). As previously observed for other RIPK1 inhibitors [50], the inhibitory potential of BHA on RIPK1 was better in cells than in vitro. Indeed, BHA completely prevented cellular RIPK1 activity and cytotoxicity when used at 100 µM (Fig. 4D, E), a concentration that is most commonly found in the literature and that was used in our cellular assays (Figs. 1–4). This cellular inhibitory effect was lost at 10 µM, but still substantial at 50 µM (Fig. 4D, E). These results identified BHA, and not BHT, as a direct RIPK1 inhibitor.

### 3-BHA docks into RIPK1 in a DLG-out/Glu-out conformation

**Fig. 5:**
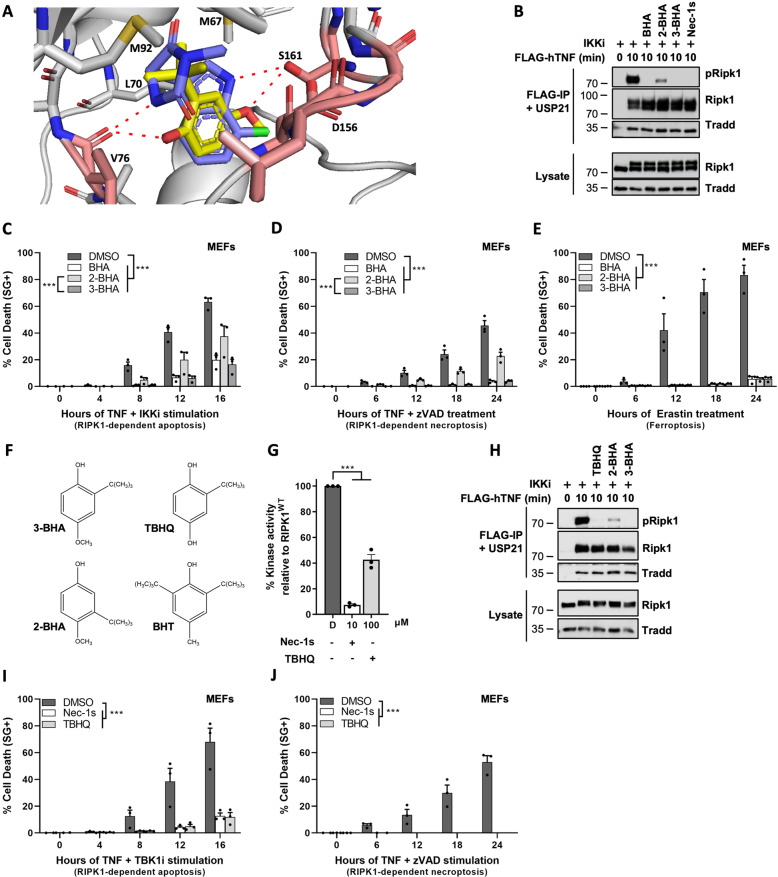
3-BHA and TBHQ function as type III inhibitors for RIPK1. **A** Docking of Nec-1s (purple) and 3-BHA (yellow) into RIPK1 (PDB: 4ITH). Hydrogen bonds (2.7–3.0 Å) with residues V76 and S161 of RIPK1 are shown as red dashes. Hydrophobic residues M67, L70, and M92 of RIPK1 engaged in hydrophobic interaction are also indicated. **B**, **H** MEFs were pretreated for 30 min with indicated compounds before stimulation with 1 µg/ml FLAG-hTNF for the indicated duration. TNFR1 complex I was FLAG-immunoprecipitated and the IPs were treated with USP21 post-IP when indicated. The results are representative of at least two independent experiments. pRIPK1 refers to autophosphorylation of RIPK1 on S166/T169. **C**–**E, I**–**J** MEFs were pretreated for 30 min with indicated compounds (100 µM BHA, 100 µM 2-BHA, 100 µM 3-BHA, 100 µM TBHQ, 10 µM Nec-1s, 10 µM GSK8612 (TBK1i), 50 µM zVAD-fmk) before stimulation with 20 ng/ml hTNF (**C**–**D, I**–**J**) or 10 µM erastin (**E**) for the indicated duration. Cell death was measured over time by Sytox Green (SG+) positivity, and the results are presented as mean ± SEM of three independent experiments (*n* = 3). Statistical analysis on kinetic cell death assays with more than one timepoint is detailed in the Methods section. **F** Molecular structure models are presented for 2-, 3-BHA, BHT, and THBQ. **G** Kinase activity was quantitatively measured by ATP consumption using the ADP-Glo kinase assay, compounds were used at indicated concentrations. D is short for DMSO. Results are presented as a percentage relative to the activity of the kinase in absence of inhibitor and are the mean ± SEM of three independent kinase assays (*n* = 3). Statistical significance was determined via ordinary one-way ANOVA followed by a Tukey post hoc test. Significance between samples is indicated in the figures as follows: *P < 0.05; **P < 0.01; ***P < 0.001; NS, not significant.

Commercial BHA is supplied as an isomeric mixture of 2- and 3-BHA (respectively, representing ~15% and 85%). Interestingly, we found that 3-BHA, but not 2-BHA or BHT, docks into an existing crystal structure of RIPK1 (PDB: 4ITH) in which Nec-1s is bound to RIPK1 in a DLG-out/Glu-out conformation, a typical feature of RIPK1 type III inhibitors but, to our knowledge, that has not been reported yet for other kinases (Fig. 5A) [51, 52]. Similarly, as Nec-1s, 3-BHA is capable of hydrogen bonding with V76 and S161 of RIPK1 in the DLG-out conformation (Fig. 5A). In addition, both Nec-1s and BHA engage in hydrophobic interactions with the hydrophobic residues M67, L70, and M92 of RIPK1, which is possible for BHA thanks to the tert-butyl group in the 3-position (Fig. 5A). The fact that such hydrophobic interactions would not be possible with the tert-butyl group in the 2-position may explain why 2-BHA could not dock into this crystal structure of RIPK1. In line with these predictions, we confirmed the superior inhibitory capacity of 3-BHA over 2-BHA, both in kinase assays (SFig. 3) and in cells, using RIPK1 activation in TNFR1 complex I (Fig. 5B) and RIPK1 kinase-dependent apoptosis (Fig. 5C) and necroptosis (Fig. 5D) as readouts. In contrast, the two BHA isomers showed similar potency in inhibiting erastin-induced ferroptosis, confirming their comparable ROS-scavenging capacities (Fig. 5E).

The structural insights on the binding of 3-BHA to RIPK1 also provided an explanation for the lack of inhibition of RIPK1 by BHT. Antioxidant BHT lacks the oxygen in the 4-position of the benzene ring that is required for hydrogen bonding with S161 of RIPK1 (Fig. 5A, Fig. 5F). Furthermore, the extra tert-butyl group in the 5-position makes BHT significantly more sterically bulky than BHA, resulting in an inability for BHT to bind to RIPK1. In contrast, the structural similarities between 3-BHA and TBHQ (oxygens in the 1- and 4-position for the respective hydrogen bonding with S161 and V76 of RIPK1, and tert-butyl group in the 3-position for hydrophobic interactions with RIPK1) suggested that TBHQ could also function as a direct inhibitor for RIPK1 (Fig. 5F). This prediction was confirmed by in vitro kinase assays using recombinant RIPK1, where ~60% reduction in RIPK1 enzymatic activity was observed at 100 µM of TBHQ (Fig. 5G). Inhibition of RIPK1 by TBHQ was further validated in cells, with TBHQ pretreatment completely preventing activation of RIPK1 in TNFR1 complex I (Fig. 5H), and TNF-mediated RIPK1 kinase-dependent apoptosis (Fig. 5I) and necroptosis (Fig. 5J).

Together, these results provided a structural basis for the direct inhibition of RIPK1 by 3-BHA, which led to the identification of the structurally related antioxidant and food additive TBHQ as an additional type III RIPK1 inhibitor. The fact that 2-BHA partially affected RIPK1 kinase activity despite its inability to bind RIPK1 in silico reveals some flexibility in the kinase domain of RIPK1 that still allows 2-BHA docking, albeit less efficiently than 3-BHA.

### Oral administration of BHA protects mice from TNF-induced lethal shock

**Fig. 6:**
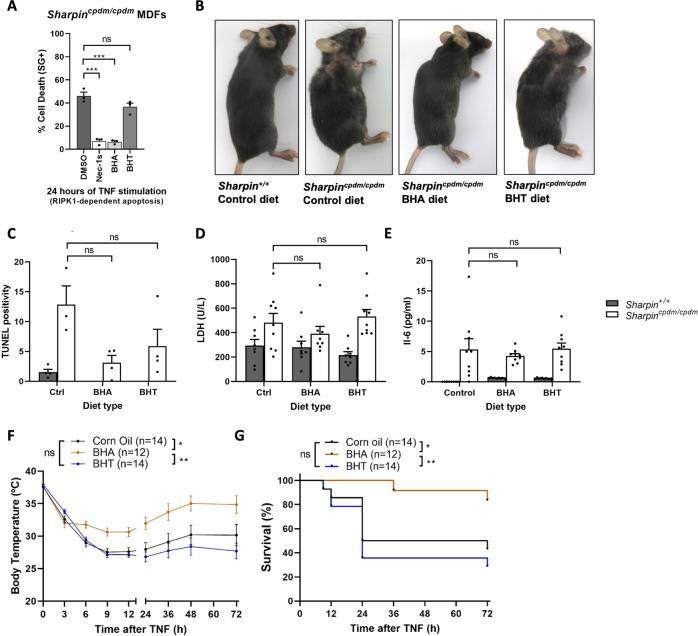
Oral administration of BHA protects mice from TNF-induced lethal shock. **A** *Sharpin*^*cpdm/cpdm*^ MDF cells were pretreated for 30 min with indicated compounds (Nec-1s 10 µM, BHA 100 µM, BHT 100 µM) before stimulation with 1 ng/ml hTNF for the indicated duration. Cell death was measured over time by Sytox Green (SG + ) positivity, and the results are presented as mean ± SEM of three independent experiments (*n* = 3). Statistical significance was determined via one-way ANOVA followed by a Tukey post hoc test. **B**–**E** 4-weeks old *Sharpin*^*+/+*^ and *Sharpin*^*cpdm/cpdm*^ littermate mice were fed for 5 weeks on a control diet or on a diet enriched in BHA or BHT. **B** A picture of the mice was taken at the end of the feeding period. **C** TUNEL quantification was performed on liver sections from 3–4 mice per diet condition. **D**–**E** Serum levels of lactate dehydrogenase (LDH) (*Sharpin*^*+/+*^: control diet *n* = 8, BHA diet *n* = 8, BHT diet *n* = 8; *Sharpin*^*cpdm/cpdm*^: control diet *n* = 9, BHA diet *n* = 9, BHT diet *n* = 9) (**D**) and IL-6 (*Sharpin*^*+/+*^: control diet *n* = 8, BHA diet *n* = 9, BHT diet *n* = 8; *Sharpin*^*cpdm/cpdm*^: control diet *n* = 9, BHA diet *n* = 9, BHT diet *n* = 9) (**E**) were determined at the end of the feeding period. **C**–**E** Statistical significance between *Sharpin*^*cpdm/cpdm*^ mice was determined via ordinary one-way ANOVA followed by a Tukey post hoc. **F**–**G** C57BL/6 J female mice were administered 100 µl pure corn oil or 100 µl corn oil containing 625 mg/kg BHA or BHT via oral gavage after 16 h of starvation. Food was re-introduced 30 min after gavage and hTNF was injected 1 h after the oral gavage at 15 µg hTNF per 20 g of body weight. Body temperature (**F**) and cumulative survival rates (**G**) were determined over time. The number of mice used in each condition is indicated. The temperature results are represented as mean ± SEM. Statistical significance of the temperature curves was determined using two-way ANOVA followed by a Tukey post hoc test. Survival curves were compared using the log-rank Mantel–Cox test. Significance between samples is indicated in the figures as follows: *P < 0.05; **P < 0.01; ***P < 0.001; NS, not significant.

As BHA and TBHQ are commonly used food additives, we next examined the effect of the oral administration of BHA on inflammatory conditions originating from TNF-mediated RIPK1 kinase-dependent cell death. We first used a chronic model of disease caused by SHARPIN deficiency in mice. These animals develop a severe multi-organ inflammatory condition, known as chronic proliferative dermatitis in mice (cpdm), resulting from tissue-specific induction of TNF/TNFR1-mediated RIPK1 kinase-dependent apoptosis or necroptosis [29]. MDFs isolated from the *Sharpin*^*cpdm/cpdm*^ mice succumb by RIPK1 kinase-dependent apoptosis upon single TNF stimulation, and we found that BHA, but not BHT, could protect these cells from death (Fig. 6A). We therefore next tested the effects of feeding the *Sharpin*^*cpdm/cpdm*^ mice with a BHA-containing diet for a period of 5 weeks, starting at 4-weeks of age, before the onset of symptoms. Interestingly, we observed that the mice fed with BHA looked macroscopically better than the ones receiving a standard diet or a diet enriched with BHT (Fig. 6B). For instance, the BHA-fed mice did not show signs of skin lesion characteristic of the Sharpin^cpdm^ mutation (Fig. 6B). Nevertheless, closer histological analysis of several organs, including not wounded skin, did not reveal a statistically significant reduction in the number of tissue-associated dead cells or immune infiltrates (Fig. 6C and data not shown). Also, the reduction in the levels of LDH and IL-6 detected in the serum of the BHA-fed mice did not reach statistical significance (Fig. 6D, E). So, while inhibition of RIPK1 by BHA could prevent TNF cytotoxicity in *Sharpin*^*cpdm/cpdm*^ MDFs, the oral administration of BHA to the *Sharpin*^*cpdm/cpdm*^ mice only marginally protected them from cell death-driven inflammation.

Next, we evaluated the potential protective effect of BHA in the acute model of systemic inflammatory response syndrome (SIRS) caused by intravenous injection of TNF, a lethal shock model previously demonstrated to originate from RIPK1 kinase-dependent cell death [53, 54]. Remarkably, oral administration of BHA significantly protected the mice from TNF-induced hypothermia and lethality (Fig. 6F, G). Importantly, the protection provided by BHA resulted from RIPK1 inhibition, and not ROS scavenging, since BHT had no effect in this acute model of disease (Fig. 6F, G).

## Discussion

Even though compelling evidence exists for the generation of ROS upon TNF sensing and during necroptosis induction [4, 7, 27, 28, 55], it has remained unclear whether these radicals are required for the execution of this cell death modality [29, 30]. On the one hand, the great protection obtained by the widely used ROS scavenger BHA supports the role of ROS for TNF-mediated necroptosis [4, 5, 7]. On the other hand, the superior anti-necroptotic potential of BHA over other antioxidants instead suggests a ROS-independent protective function of BHA [4, 32–34]. In this study, we made the surprising finding that BHA functions as a direct RIPK1 inhibitor, and demonstrated that the protective role of BHA against TNF cytotoxicity solely originates from this newly identified function of BHA. Indeed, out of a panel of seven different antioxidants, BHA was the only one providing significant protection against necroptosis. Furthermore, we found that BHA also protected cells from apoptosis, but that the anti-death potential of BHA was limited to forms of necroptosis and apoptosis relying on RIPK1 kinase activity. We, therefore, conclude from these experiments that ROS is dispensable for TNF-mediated necroptosis. We can, however, not exclude a possible cell-specific involvement of ROS, since antioxidants other than BHA were reported to protect other cell types, such as Jurkat cells, from TNF-mediated necroptosis [31]. Nevertheless, these results may need to be interpreted with caution, as an effect on cell death does not necessarily mean a role for ROS in the cell death execution phase. Indeed, we found that the reported protection provided by Rotenone and NDGA against TNF-induced necroptosis was in fact indirect and most probably owing to defective TNFR1 complex I assembly. But our results also contrast with previous studies performed in the same cells. It was indeed reported that the sensing of ROS on three distinct cysteine residues of RIPK1 is required for its enzymatic activation and pro-necroptotic potential in L929 cells [26]. Our results instead show that BHA, but not BHT, protects L929 cells from TNF-mediated RIPK1 kinase-dependent necroptosis, excluding a major role of ROS for RIPK1 cytotoxicity in these cells. We may therefore question whether the protection observed in cells expressing the three cysteine-mutated versions of RIPK1 really reflects a need for ROS and not a defect in RIPK1 tertiary structure, which could affect its ability to induce necroptosis independently of an oxidation impairment.

The IC_50_ value of BHA for RIPK1 was estimated to be ~100 µM by in vitro kinase assays making use of recombinant RIPK1, but approximately three times lower when looking at endogenous RIPK1 activity in cells. This difference may be explained by the fact that BHA is a strongly lipophilic compound that will cluster at the membrane compartments of the cell, possibly locally enriching its effective concentration. It is important to note that BHA is most commonly used at 100 µM for its antioxidant properties in cells, a concentration at which BHA completely prevents cellular activity and cytotoxicity of RIPK1. This implies that the reported cellular effects of BHA found in the literature, either within or outside the cell death field, may in fact originate from RIPK1 kinase inhibition rather than ROS scavenging, a possibility that will require further investigation. Commercial BHA is supplied as a mixture of two isomers, with 3- BHA accounting for the vast majority. Our in silico analysis predicted binding of 3-BHA, but not of 2-BHA or BHT, to RIPK1 in an inactive DLG-out/Glu-out conformation, similar to the binding of the type III inhibitor Nec-1s to RIPK1 (PDB: 4ITH). In contrast, none of these compounds were able to dock into RIPK1 in a DLG-out/Glu-in conformation, as observed for a type II 1-aminoisoquinoline inhibitor (PDB: 4NEU). These structural predictions were experimentally validated in cells, with 3-BHA showing higher inhibitory capacity on RIPK1 than 2-BHA. The fact that 2-BHA still partially affected RIPK1 kinase activity despite its predicted inability to bind RIPK1 highlights the limitation of these in silico analyses, which do not account for the plasticity that protein structures can show in solution. Nevertheless, the structural insights on the binding of 3-BHA to RIPK1 led to the identification of the structurally related synthetic antioxidant TBHQ as an additional type III RIPK1 inhibitor.

As BHA and TBHQ are commonly used food additives, we examined the possibility that oral administration of BHA, but not BHT, would provide protection to acute and chronic inflammatory conditions resulting from TNF-mediated RIPK1 kinase-dependent cell death. Our results demonstrated a ROS-independent, but RIPK1 kinase-dependent, the effect of BHA in the acute model of TNF-induced SIRS, where its oral administration protected mice from TNF-mediated RIPK1 kinase-dependent hypothermia and lethality. These results are in line with a previous study reporting protection by BHA against the combined injection of TNF and zVAD-fmk, another RIPK1 kinase-dependent lethal trigger [56]. In contrast, oral administration of BHA had little effect in the chronic model of TNF-driven inflammatory disease that we tested. SHARPIN deficiency in mice causes a severe multi-organ inflammatory pathology, known as chronic proliferative dermatitis (cpdm), which results from TNF/TNFR1-mediated RIPK1 kinase-dependent apoptosis and necroptosis. We found that feeding the *Sharpin*^*cpdm/cpdm*^ mice a BHA-containing diet only marginally reduced their inflammatory phenotype. Although these results contrast with the complete protection obtained by genetic inactivation of RIPK1 kinase activity [57], they are in accordance with a recent study reporting absence of protection when feeding the *Sharpin*^*cpdm/cpdm*^ mice GNE684, a potent RIPK1 inhibitor [58]. Together, these results, therefore, indicate that the pathology in this mouse model is too severe to obtain therapeutic benefit by pharmacological inhibition of RIPK1, possibly due to problems of bioavailability and distribution. This is, however, not the case in another model of chronic inflammation driven by RIPK1 kinase-dependent cell death. NEMO deficiency in intestinal epithelial cells (IECs) is reported to cause RIPK1 kinase-dependent colitis and ileitis [59], and oral administration of GNE684 was shown to afford dose-dependent protection from IEC death and associated inflammation in these mice [58]. It would therefore be interesting to evaluate whether oral administration of BHA also provides such a therapeutic advantage to the NEMO^IEC-KO^ mice. In support of this idea, it was previously reported that NEMO deletion in liver parenchymal cells (LPCs) leads to steatohepatitis and hepatocellular carcinoma and that feeding the NEMO^LPC-KO^ mice a BHA-containing diet completely prevented disease development [60]. The protection conferred by BHA in this liver model most probably originates from RIPK1 inhibition, and not ROS-scavenging, as crossing the NEMO^LPC-KO^ mice with the RIPK1 kinase-dead mice was recently reported to also completely rescue their phenotype [61]. It is important to note that these feeding experiments were performed with a standard diet enriched with 0.7% w/w BHA, a concentration that is 35-times higher than the maximal 0.02% allowed in the food intended for human consumption. It is therefore unlikely that the daily ingestion of BHA by humans would suffice for systemic inhibition of RIPK1. It is important to mention that diets with 0.7% BHA are commonly used by the scientific community to claim implications of ROS in a wide variety of mouse models. Our results now clearly question the conclusions from these studies, as the reported effects of BHA in these in vivo experiments may instead originate from RIPK1 kinase inhibition.

In conclusion, our study led to the surprising discovery that BHA and TBHQ function as two new types III RIPK1 inhibitors, implying that they should no longer be used as strict antioxidants. These unexpected findings highlight the risk of misinterpretation in previous studies using these compounds for their antioxidant properties, which may consequently also lead to the identification of new biological processes controlled by RIPK1 enzymatic activity.

## Supplementary information

Supplementary information

## Data Availability

All data generated or analyzed during this study are included in this published article [and its supplementary information files].
