# Supplementary material for: Antioxidant and food additive BHA prevents TNF cytotoxicity by acting as a direct RIPK1 inhibitor
Source: Cell Death Dis. 2021 Jul 14;12(7):699. doi: 10.1038/s41419-021-03994-0 (PMC8280128; doi:10.1038/s41419-021-03994-0)
Supplement: Supplementary file 1 — Supplementary information [file 41419_2021_3994_MOESM1_ESM.docx]

**Supplementary information**

**
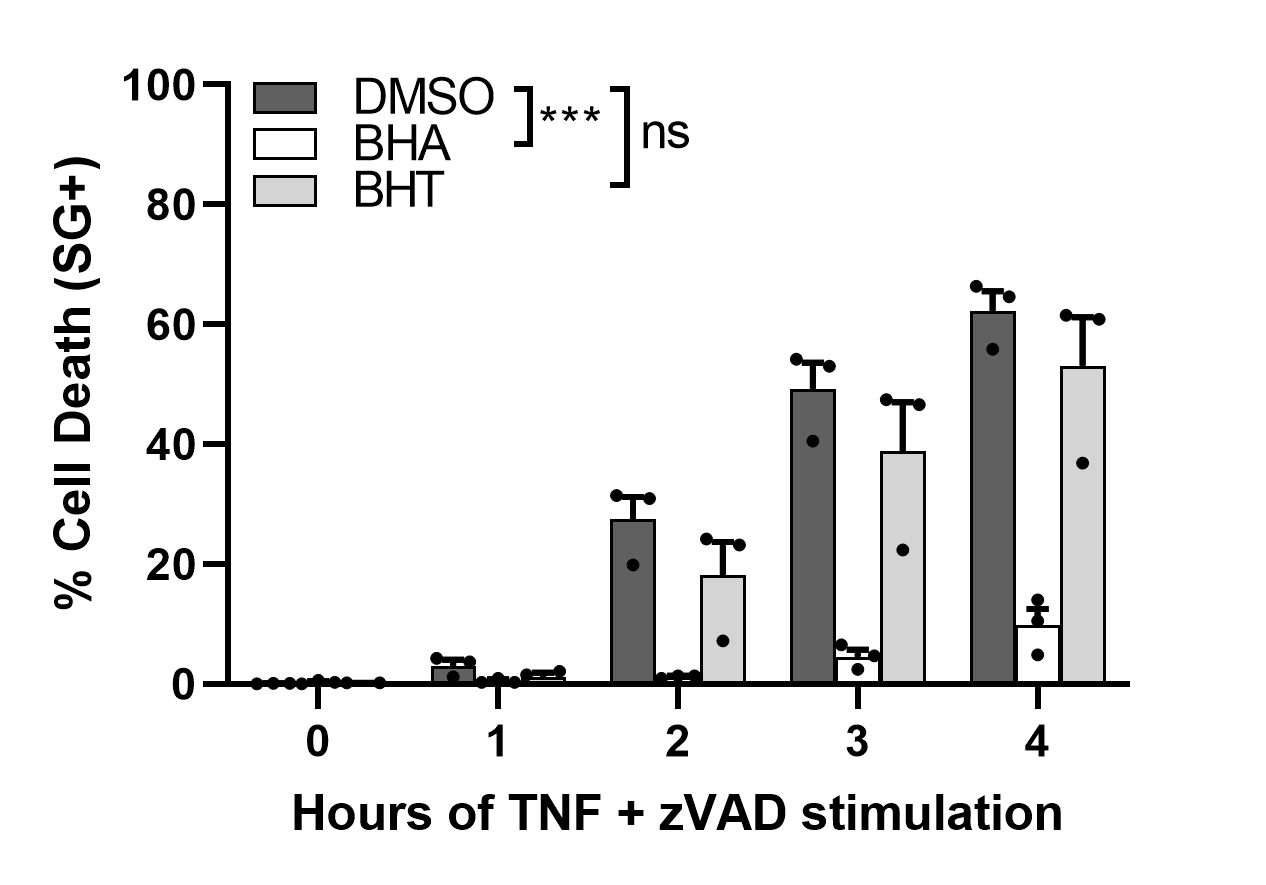

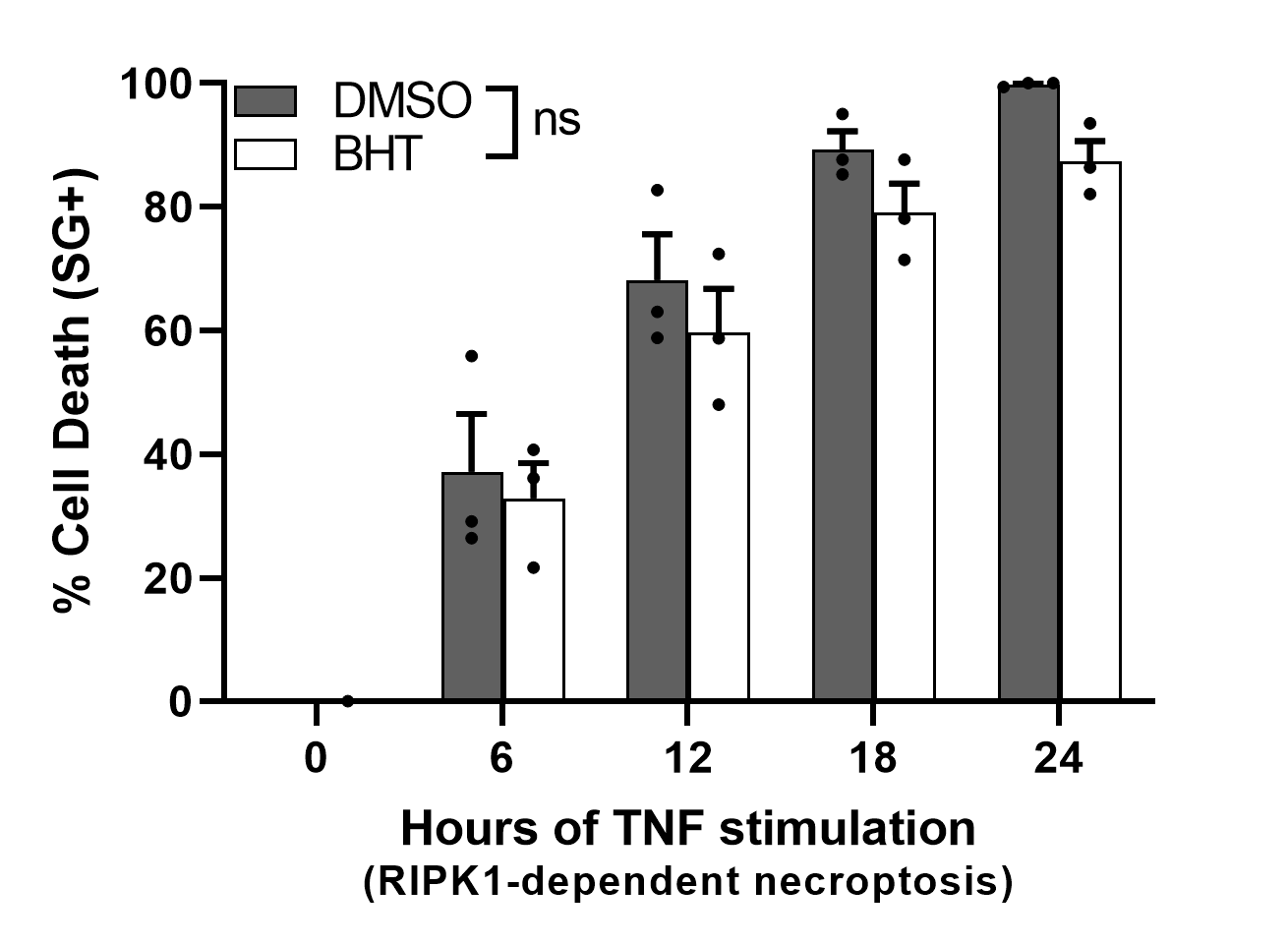
**

**Supplementary Figure 1, related to Figure 2**

**B**

**L929s**

**A**

**MDFs**

**Supplementary Figure 1. The protective role of BHA is independent of its ROS-scavenging properties.** (**A**-**B**) MDFs (**A**) and L929 cells (**B**) were pretreated for 30min with indicated compounds (100µM BHA, 100µM BHT, 50µM zVAD) before stimulation with 20ng/ml hTNF for the indicated duration. Cell death was measured over time by Sytox Green (SG+) positivity. Cell death assays are presented as mean ± SEM of 3 independent experiments (n=3). Statistical analysis on kinetic cell death assays with more than one timepoint is detailed in the Methods section. Significance between samples is indicated in the figures as follows: * P<0.05; ** P<0.01; *** P<0.001; NS, not significant.

**Supplementary Figure 2, related to Figure 3**

**A**

**B**

**C**

**D**

**Supplementary Figure 2. BHA prevents cellular activation of RIPK1.** (**A**-**D**) MEFs (**A**-**B**), HT-29 cells (**C**) and BT549 cells (**D**) were pretreated for 30min with indicated compounds before stimulation with 1µg/ml FLAG-hTNF for the indicated duration. TNFR1 complex I was then FLAG-immunoprecipitated and the IPs were treated with USP21 before analysis by immunoblot. pRIPK1 refers to autophosphorylation of RIPK1 on S166. The results are representative of at least 2 independent experiments.


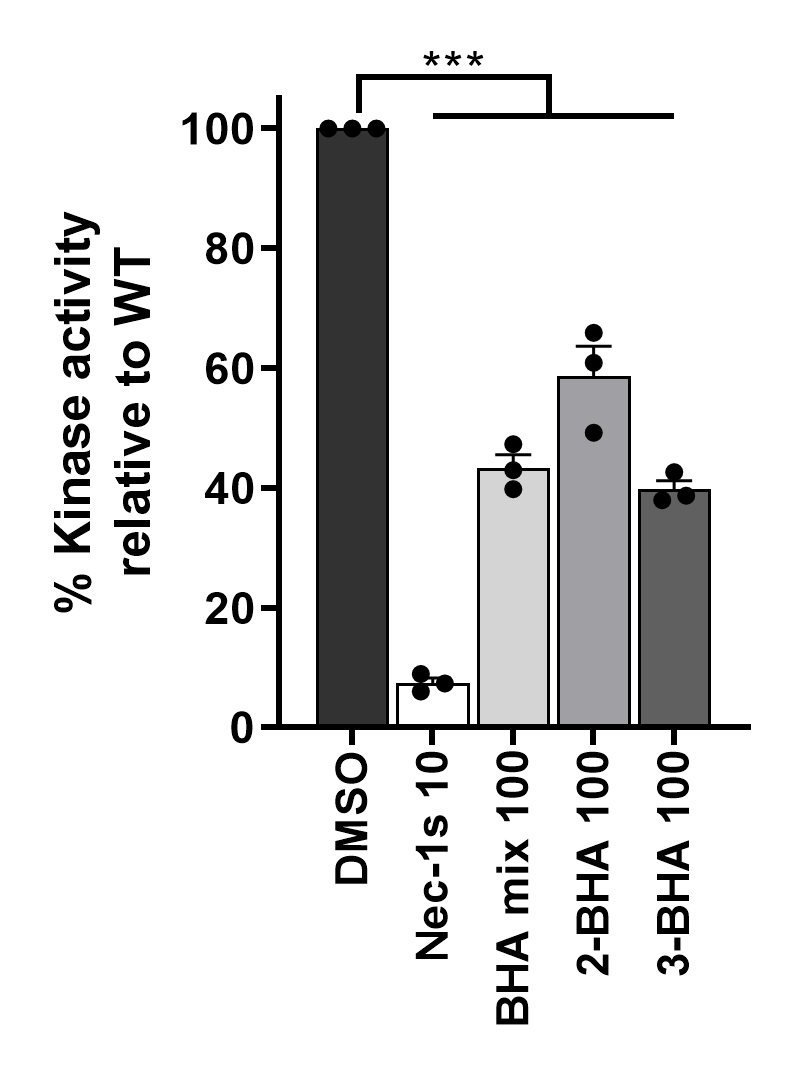


**Supplementary Figure 3, related to Figure 5**


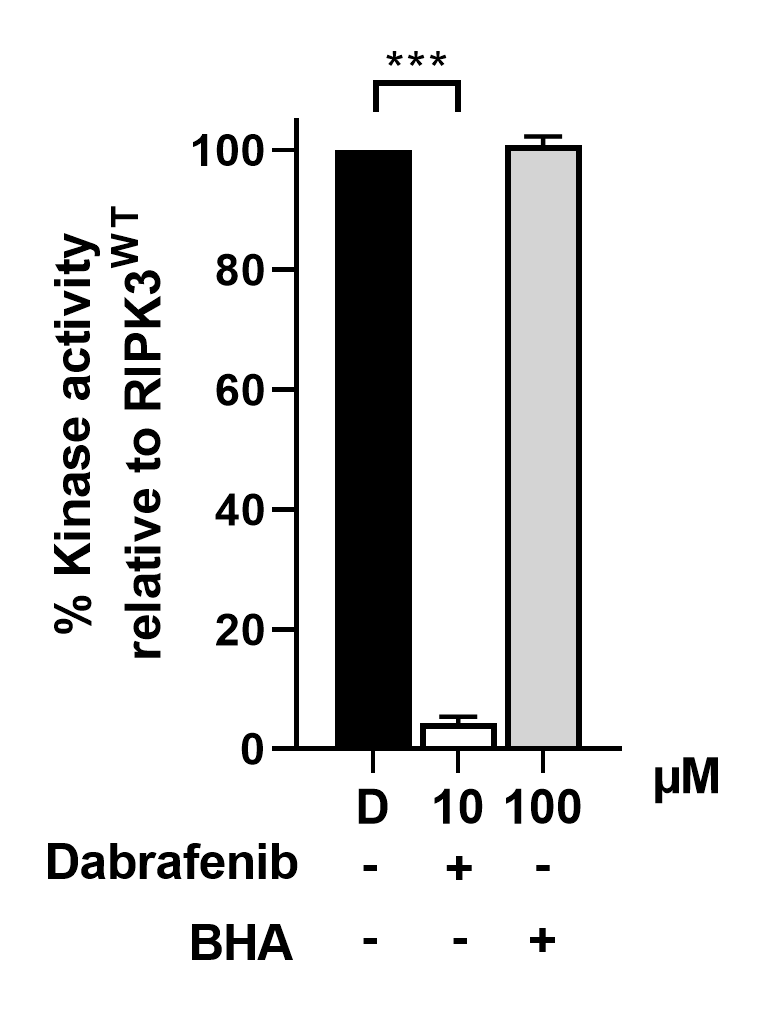


**Supplementary Figure 3. 3-BHA and TBHQ function as a type III inhibitors for RIPK1.** RIPK1 (AA 1–479) kinase activity was quantitatively measured by ATP consumption using the ADP-Glo kinase assay, and the compounds were used at indicated concentrations. Results are presented as a percentage relative to the activity of RIPK1 in absence of inhibitor, and is the mean ± SEM of 3 independent kinase assays (n = 3). Statistical significance was determined via ordinary one-way ANOVA followed by a Tukey post-hoc test. Significance between samples is indicated in the figures as follows: * P<0.05; ** P<0.01; *** P<0.001; NS, not significant.
